# Supplementary material for: Berberine-Encapsulated Poly(lactic-co-glycolic acid)–Hydroxyapatite (PLGA/HA) Microspheres Synergistically Promote Bone Regeneration with DOPA-IGF-1 via the IGF-1R/PI3K/AKT/mTOR Pathway
Source: Int J Mol Sci. 2023 Oct 20;24(20):15403. doi: 10.3390/ijms242015403 (PMC10607899; doi:10.3390/ijms242015403)

Supplementary materials for

Berberine-Encapsulated Poly(lactic-co-glycolic acid)–Hydroxyapatite (PLGA/HA) Microspheres Synergistically Promote Bone Regeneration with DOPA-IGF-1 via the IGF-1R/PI3K/AKT/mTOR Pathway

Li Chen [1], Meng Tian [1], Jing Yang [1] and Zhenxu Wu [2,*]

[1] Key Laboratory of Molecular Epigenetics of Ministry of Education, Institute of Cytology and Genetics, Northeast Normal University, 5268 Renmin Street, Changchun 130024, China; chenl166@nenu.edu.cn (L.C.); tianm132@nenu.edu.cn (M.T.); yangj357@nenu.edu.cn (J.Y.)

[2] Key Laboratory of Polymer Ecomaterials, Changchun Institute of Applied Chemistry, Chinese Academy of Sciences, 5625 Renmin Street, Changchun 130022, China

* Correspondence: wzx131@ciac.ac.cn; Tel.: +86-0431-8526-2390

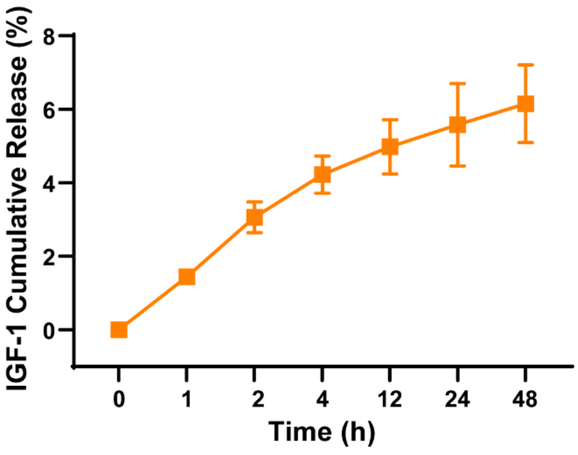

Figure S1. IGF-1 (100 ng) cumulative release profiles for 0, 1, 2, 4, 12, 24 and 48 h.

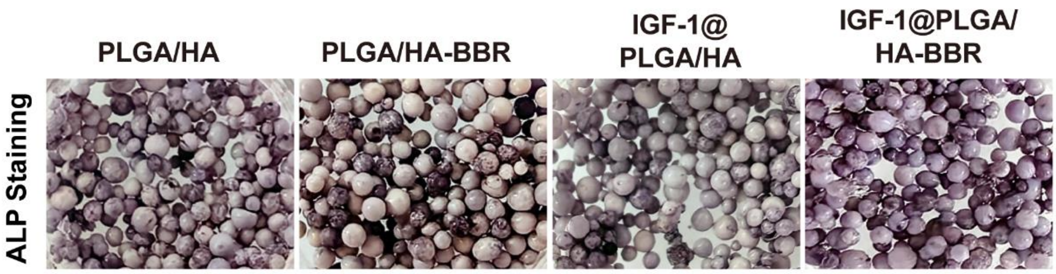

Figure S2. ALP staining of MC3T3-E1 cells on different sets of microspheres (7 days).

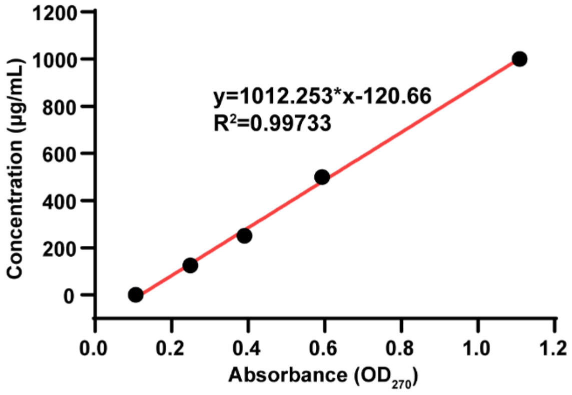

Equation	y=a + b*x		
Weight	No Weighting		
Residual Sum of Squares	1249.78		
Pearson's r	0.999		
Adj. R-Square	0.997		
		Value	Standard Error
B	Intercept	-120.660	15.729
	Slope	1012.253	26.160

Figure S3. Standard curve for determination of BBR solution concentrations. The standard curve is plotted using the absorbance values of the BBR solution at 270 nm for different concentrations. On the left is the diagram of the standard curve and on the right are the specific parameters of the curve.

Table S1. Detection of adhesion efficiency of IGF-1 (100 ng).

Initial amout	adhesion amount (ng)	Non-adhesion amount (ng)	Adhesion efficiency
100	90.28 ± 0.18	9.722 ± 0.176	90.278%

Table S2. Sphericity of microspheres.

Sample	Sphericity
PLGA/HA	0.9630 ± 0.0168
PLGA/HA-BBR	0.9551 ± 0.0359
IGF-1@PLGA/HA	0.9527 ± 0.0344
IGF-1@PLGA/HA-BBR	0.9517 ± 0.0218

Table S3. Primer information for RT-PCR.

Gene	Forward primer sequence (5'-3')	Reverse primer sequence (3'-5')
Col1	CCCAGCGGTGGTTATGACTT	TCGATCCAGTACTCTCCGCT
Runx2	GCCAGTAATCTTCGTGCCAG	TAGTGAGCTTCTTCCTGGGGA
*Spp1(*OPN*)*	CCAGCCAAGGACCAACTACA	AGTGTTTGCTGTAATGCGCC
*Bglap(*OCN*)*	AAGCAGGAGGGCAATAAGGT	TTTGTAGGCGGTCTTCAAGC
Gapdh	CTTGTGCAGTGCCAGCCTC	GATGGTGATGGGTTTCCCGT

Table S4. Information of antibody used in this study.

Antibody Name	Manufacturer	Cat#	Source/Isotype	MW (kDa)
p-mTOR (Ser2448)	Cell Signaling	5536	Rabbit	289
mTOR	Cell Signaling	2983	Rabbit	289
p-AKT (S473)	Cell Signaling	9271S	Rabbit	60
AKT	Cell Signaling	9272S	Rabbit	60
IGF1R	Invitrogen	MA5-13817	Rabbit	155
β-Actin	Abclonal	AC026	Rabbit	42

Full Western Blots

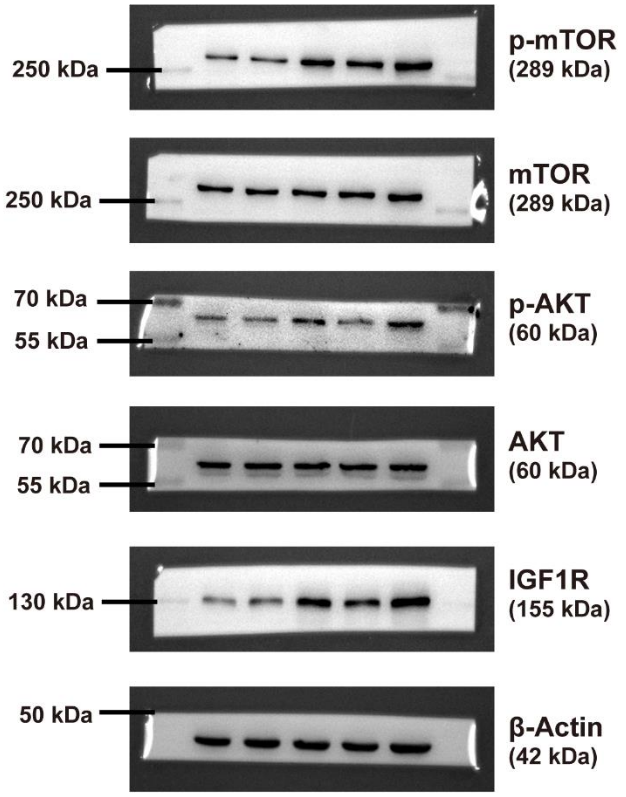

Supplement: Supplementary file 1 [file ijms-24-15403-s001.zip › ijms-2637168-supplementary.pdf]
